# Supplementary material for: A Longitudinal Analysis of Alcohol Use Behavior among Korean Adults and Related Factors: A Latent Class Growth Model
Source: Int J Environ Res Public Health. 2021 Aug 20;18(16):8797. doi: 10.3390/ijerph18168797 (PMC8391448; doi:10.3390/ijerph18168797)
Supplement: Supplementary file 1 [file ijerph-18-08797-s001.zip › ijerph-1310673-supplementary.pdf]

Table S1. General Characteristics according to Each Class (N=8267)

Characteristics	Categories	Moderate to low risk class (Class 1, 7.31%) n (%)	Low to moderate risk class (Class 2, 5.93%) n (%)	Stable moderate risk class (Class 3, 13.25%) n (%)	Stable low risk Class (Class 4, 73.52%) n (%)	Total	χ^2	*p*
Age(yrs)	19-29	18 (3.0)	129 (26.3)	38 (3.5)	930 (15.3)	1115 (13.5)	315.033	<.001
	30-39	98 (16.2)	127 (25.9)	238 (21.7)	1394 (22.9)	1857 (22.5)		
	40-49	207 (34.3)	133 (27.1)	440 (40.2)	1817 (29.9)	2597 (31.4)		
	50-60	281 (46.5)	101 (20.6)	379 (34.6)	1937 (31.9)	2698 (32.6)		
Gender	Male	521 (86.3)	344 (70.2)	984 (89.9)	2145 (35.3)	3994 (48.3)	1612.045	<.001
	Female	83 (13.7)	146 (29.8)	111 (10.1)	3933 (64.7)	4273 (51.7)		
Occupation	Unemployed	113 (18.7)	92 (18.8)	114 (10.4)	2013 (33.1)	2332 (28.2)	292.099	<.001
	Employed	491 (81.3)	398 (81.2)	981 (89.6)	4065 (66.9)	5935 (71.8)		
Type of family	Intact families	560 (92.7)	416 (84.9)	979 (89.4)	5632 (92.7)	7587 (91.8)	55.176	<.001
	Single-person families	41 (6.8)	63 (12.9)	105 (9.6)	363 (6.0)	572 (6.9)		
	Grandparent/single-parent families	3 (0.5)	11 (2.2)	11 (1.0)	83 (1.4)	108 (1.3)		
Level of education	High school graduates or below	353 (58.4)	274 (55.9)	605 (55.3)	3345 (55.0)	4577 (55.4)	2.652	.448
	College graduates or above	251 (41.6)	216 (44.1)	490 (44.7)	2733 (45.0)	3690 (44.6)		
Household income	Low-income	524 (86.8)	437 (89.2)	984 (89.9)	5256 (86.5)	7201 (87.1)	11.509	.009
	Regular-income	80 (13.2)	53 (10.8)	111 (10.1)	822 (13.5)	1066 (12.9)		

Table S2. The number of subjects were evaluated in each wave (N=8267)

Wave	Year	n	%
1	2009	6,149	74.4
2	2010	6,259	75.7
3	2011	6,298	76.2
4	2012	7,399	89.5
5	2013	7,284	88.1
6	2014	7,074	85.6
7	2015	6,900	83.5
8	2016	6,633	80.2
9	2017	6,437	77.9
10	2018	6,206	75.1
11	2019	5,960	72.1
